# Suspicious amorphous microcalcifications detected on full-field digital mammography: correlation with histopathology

**DOI:** 10.1590/0100-3984.2017.0025

**Published:** 2018

**Authors:** Vera Christina Camargo de Siqueira Ferreira, Elba Cristina Sá de Camargo Etchebehere, José Luiz Barbosa Bevilacqua, Nestor de Barros

**Affiliations:** 1 MD, PhD, Hospital Sírio Libanês and Instituto do Câncer do Estado de São Paulo (Icesp), São Paulo, SP, Brazil.; 2 MD, PhD, Faculdade de Ciências Médicas da Universidade Estadual de Campinas (FCM-Unicamp), Campinas, SP, Brazil.; 3 MD, PhD, Hospital Sírio Libanês, São Paulo, SP, Brazil.; 4 MD, PhD, Tenured Professor, Faculdade de Medicina da Universidade de São Paulo (FMUSP), São Paulo, SP, Brazil.

**Keywords:** Breast stereotaxis biopsy, Calcification, Diagnosis, Amorphous morphology, Digital mammography, Breast cancer, Neoplasias mamárias/diagnóstico, Mamografia digital, Biópsia por agulha, Microcalcificações mamárias, Calcificações amorfas, Câncer de mama

## Abstract

**Objective:**

To evaluate suspicious amorphous calcifications diagnosed on full-field digital mammography (FFDM) and establish correlations with histopathology findings.

**Materials and Methods:**

This was a retrospective study of 78 suspicious amorphous calcifications (all classified as BI-RADS^®^ 4) detected on FFDM. Vacuum-assisted breast biopsy (VABB) was performed. The histopathological classification of VABB core samples was as follows: pB2 (benign); pB3 (uncertain malignant potential); pB4 (suspicion of malignancy); and pB5 (malignant). Treatment was recommended for pB5 lesions. To rule out malignancy, surgical excision was recommended for pB3 and pB4 lesions. Patients not submitted to surgery were followed for at least 6 months.

**Results:**

Among the 78 amorphous calcifications evaluated, the histopathological analysis indicated that 8 (10.3%) were malignant/suspicious (6 classified as pB5 and 2 classified as pB4) and 36 (46.2%) were benign (classified as pB2). The remaining 34 lesions (43.6%) were classified as pB3: 33.3% were precursor lesions (atypical ductal hyperplasia, lobular neoplasia, or flat epithelial atypia) and 10.3% were high-risk lesions. For the pB3 lesions, the underestimation rate was zero.

**Conclusion:**

The diagnosis of precursor lesions (excluding atypical ductal hyperplasia, which can be pB4 depending on the severity and extent of the lesion) should not necessarily be considered indicative of underestimation of malignancy. Suspicious amorphous calcifications correlated more often with precursor lesions than with malignant lesions, at a ratio of 3:1.

## Abstract (pt)

**Objetivo:**

Correlacionar o achado mamográfico de calcificações amorfas suspeitas diagnosticadas na mamografia digital com seus diagnósticos anatomopatológicos.

**Materiais e Métodos:**

Setenta e oito casos de calcificações amorfas suspeitas (todas classificadas como BI-RADS^®^ 4) detectadas na mamografia digital e submetidas a biópsia percutânea assistida à vácuo foram retrospectivamente avaliados. A classificação anatomopatológica utilizada na biópsia foi: pB2 para lesão benigna, pB3 para lesão com potencial incerto de malignidade, pB4 para lesão suspeita, e pB5 para lesão considerada maligna. O tratamento foi recomendado para as lesões pB5, a exérese cirúrgica foi indicada para lesões pB3 e pB4, para descartar malignidade, e o seguimento evolutivo foi adotado para as demais pacientes.

**Resultados:**

A histologia demonstrou 8 (10,3%) casos malignos (6 lesões pB5 e 2 lesões pB4) e 36 (46,2%) casos benignos (pB2). As demais 34 (43,6%) lesões foram classificadas como pB3 (33,3% foram lesões precursoras - hiperplasia ductal atípica, neoplasia lobular ou atipia epitelial plana - e 10,3% foram lesões de alto risco). A taxa de subestimação das lesões pB3 foi zero.

**Conclusão:**

O diagnóstico de lesões precursoras (excluindo hiperplasia ductal atípica, que pode corresponder a lesão pB4 dependendo da severidade e extensão dos achados) na biópsia percutânea assistida à vácuo por calcificações amorfas suspeitas não necessariamente representa lesão subestimada. Calcificações amorfas suspeitas se associaram a lesões precursoras numa proporção de 3:1 em relação às lesões malignas.

## INTRODUCTION

Breast cancer is the leading cancer in women, in developed and developing countries^(1)^. The use of mammography enables early detection of breast cancer and leads to a reduction in mortality from the disease, as demonstrated in studies performed with conventional screen-film mammography^(2,3)^.

In the last decade, the introduction of full-field digital mammography (FFDM) for screening has yielded enhanced diagnostic benefits. Comparative studies of conventional mammography and FFDM have shown the latter to be superior in terms of the identification of microcalcifications, thus increasing detection rates for ductal carcinoma in situ (DCIS) and invasive carcinoma^(4-6)^.

In the last year, the subject of mammographic screening and percutaneous biopsies has gained visibility in editorials^(7,8)^ and articles^(9)^ published in the radiology literature of Brazil. In fact, knowledge of clinical practice is fundamental to improving patient care.

Grouped amorphous microcalcifications constitute the most discrete morphology related to suspicious calcifications detected by mammography. There is a need to understand how these findings detected by FFDM are related to the presence of DCIS and invasive carcinomas. The aim of the present study was to correlate suspicious amorphous calcifications (identified on FFDM) with histopathological findings.

## MATERIALS AND METHODS

This was a retrospective study conducted at two health care clinics operated by a private institution. The study was approved by the research ethics committee of the institution. Because of the retrospective nature of the study, informed consent was deemed unnecessary.

### Patient selection

**Figure 1:**
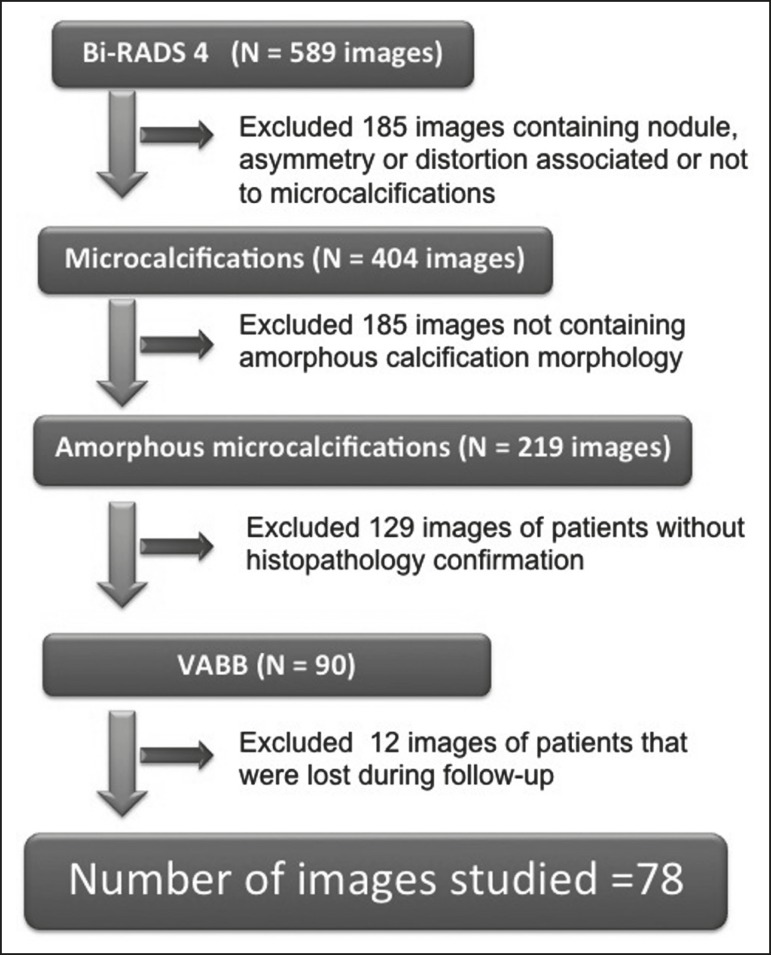
Diagram of the 589 images classified as BI-RADS category 4 findings. After application of the study criteria, 78 images, in 77 patients, were deemed eligible for evaluation.

In the first year after the introduction of the digital technique (2006 at one clinic and 2007 at the other), we reviewed all consecutive FFDM reports for female patients in whom the findings were classified as BI-RADS^®^ category 4^(10)^. The FFDM examination was performed for either screening or diagnostic purposes (in the latter case to verify findings obtained at our breast care center, which is a referral center). All biopsy samples were obtained via the vacuum-assisted breast biopsy (VABB) technique. We included patients regardless if the following clinical conditions was also present: a family history of cancer; a history of breast or ovarian cancer; previous biopsy-proven diagnosis of a precursor lesion; and bilateral suspicious lesions. We initially reviewed all FFDM images classified as BI-RADS category 4 (n = 589). Of those 589 examinations, 511 were excluded on the basis of the following criteria: the images showing a nodule, asymmetry, or distortion, with or without microcalcifications (n = 185); the images not showing an amorphous calcification morphology (n = 185); patients without histopathological analysis confirmation by VABB (n = 129); and patients having been lost to follow-up (n = 12). Therefore, the final sample comprised 78 FFDM images of suspicious amorphous calcifications, in 77 patients (Figure 1). These patients were followed-up for at least six months or underwent surgical excision of suspicious or malignant lesions.

### FFDM

Mammograms were obtained with a digital mammography system (Lorad Selenia; Hologic, Danbury, CT, USA). At least two projections, necessarily including craniocaudal and mediolateral oblique views, were obtained for analysis. True geometric (air-gap) magnification views were also obtained.

Images were displayed on a dedicated 5-megapixel display. The images were analyzed by two breast imaging radiologists, each with over 10 years of experience. In the event of disagreement, a final decision was made by a third experienced breast imaging radiologist.

**Figure 2:**
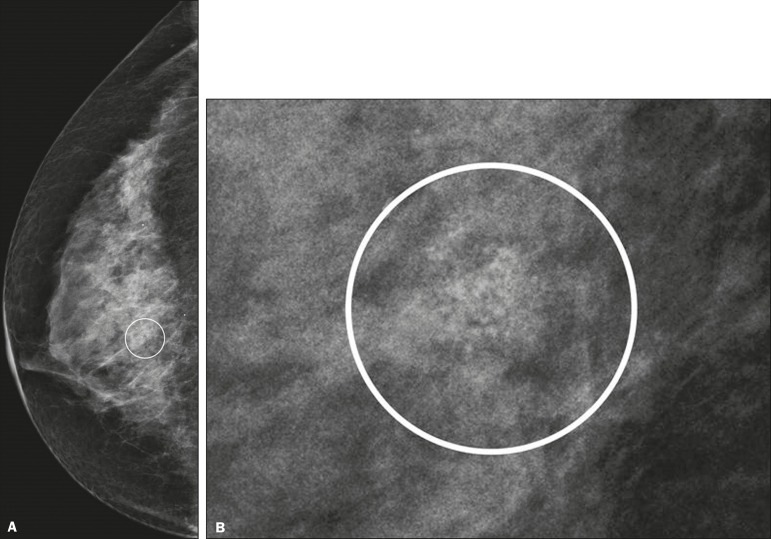
Grouped amorphous microcalcifications in a DCIS, shown in craniocaudal and magnified lateral views (**A** and **B**, respectively).

**Figure 3:**
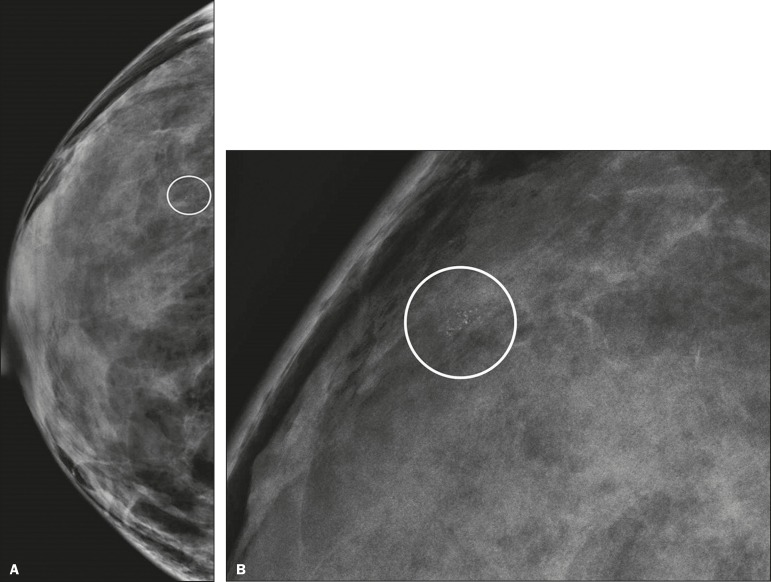
Grouped punctate and amorphous microcalcifications in a benign lesion-craniocaudal implant-displaced (Eklund) and magnified lateral views (**A** and **B**, respectively).

**Figure 4:**
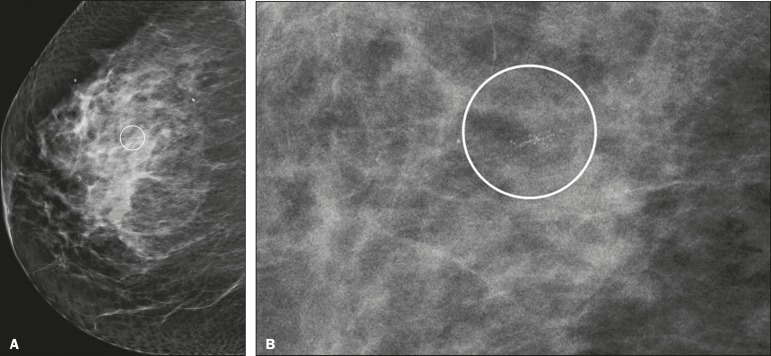
Punctate microcalcifications together with amorphous microcalcifications, with a linear distribution, in a benign lesion-mediolateral and magnified lateral views (**A** and **B**, respectively).

The suspicious amorphous microcalcifications (all classified as BI-RADS category 4 findings) showed the following morphologic characteristics: solely amorphous (Figure 2); or punctate and amorphous (Figure 3). In addition, the distribution of the microcalcifications was classified as grouped, linear (Figure 4), or segmental. There were no cases of microcalcifications with a regional distribution.

### VABB

Core biopsies were performed on a prone biopsy table (Multicare; Hologic) with a VABB system (Mammotome; Ethicon Endo-Surgery, Juarez, Mexico) and an 11-gauge needle. To assist the pathologist, radiographs of all cores were obtained. Two separate core vials-one containing microcalcifications and one containing other fragments-were sent to the pathology laboratory, together with their corresponding radiographs. At the end of the procedure, the biopsy site was marked with an identifying clip.

In all patients, craniocaudal and lateral radiographs were acquired after the procedure. Those radiographs were obtained for the following reasons: to confirm the removal of the lesion and the placement of the identifying clip; to guide the preoperative localization when surgical excision was required; and to establish a baseline for use in patient follow-up.

### Histopathological findings

All histopathological analyses were performed in a pathology laboratory, by a pathologist with over 11 years of experience in evaluating breast lesions. The classification of the histopathological diagnoses of lesions submitted to diagnostic VABB were based on the guidelines for non-operative diagnostic procedures and reporting in breast cancer screening established by the United Kingdom National Coordinating Committee for Breast Pathology^(11)^: B1, normal; B2, benign; B3, uncertain malignant potential; B4, suspicion of malignancy; and B5, malignant. Hereafter, each of those pathological categories will be preceded by the letter "p" to avoid confusion with the BI-RADS categories. Therefore, a diagnosis of atypical intraductal epithelial proliferation could be classified as pB3 or pB4 depending on the severity and extent of the lesion^(11)^.

In the presence of concurrent diagnoses within the same lesion, the primary diagnosis was designated as follows, in descending order by severity: invasive carcinoma; DCIS; atypical ductal hyperplasia (ADH); lobular neoplasia, comprising both lobular carcinoma in situ (LCIS) and atypical lobular hyperplasia (ALH); flat epithelial atypia (FEA); other high-risk lesions, including radial scar (RS) and papillary lesion; and benign lesions.

For statistical purposes, pB2 lesions were classified as benign, whereas pB4 and pB5 lesions were grouped together and classified as malignant. Category pB3 included ADH, LCIS, ALH, FEA, RS, and papillary lesions. Among those, ADH, LCIS, ALH, and FEA were considered precursor lesions.

### Surgical excision and patient follow-up

All pB4 and pB5 lesions were submitted to surgical excision. All benign lesions (pB2) were followed through-up with clinical evaluation and FFDM (after a minimum of 6 months). To avoid underestimation and to rule out adjacent malignancy, we recommended surgical excision for pB3 lesions. Patients who refused surgery were followed.

### Statistical analysis

For patient ages, the median and range were described. To evaluate the equivalency between the group of patients studied and the population of patients that were excluded, the nonparametric Wilcoxon rank sum test was applied to test age and the Pearson's chi-square test was applied to test the other demographic variables. Because multiple comparisons were tested (for age and demographic variables), the level of significance (*p*-value) of each separate test was divided by the number of tests performed (n = 5). The *p*-value required to indicate statistical significance, with Bonferroni correction for multiple comparisons (based on an α of 0.05), was calculated as 0.01.

## RESULTS

### Patient data

Our sample included 78 examinations from 77 patients (one with bilateral lesions). Patients ages ranged from 40 to 81 years, with a median of 53 years.

The patients enrolled underwent FFDM for screening (in 72%); because of a family history of cancer (in 21%); because of a patient history of breast or ovarian cancer (in 5%); or because of a patient history of mammary atypia (in 3%). The group of patients studied was equivalent to the population of patients that were excluded regarding age (*p* = 0.838); reason for performing FFDM (*p* = 0.015); family history of cancer (*p* = 0.033); history of cancer (*p* = 0.047); and bilateral BI-RADS category 4 findings (*p* = 0.815).

### FFDM findings

The morphologic characteristics of the suspicious amorphous microcalcifications were as follows: solely amorphous, in 53 (68.0%) of the 78 cases, and punctate microcalcifications accompanied by amorphous microcalcifications, in 25 (32.1%). Among the 78 cases, the distribution of the suspicious amorphous microcalcifications was classified as grouped in 66 (84.6%), as linear in 8 (10.2%), and as segmental in 4 (5.1%). Of the 12 microcalcifications with linear or segmental distribution, 6 (50.0%) showed a punctate/amorphous morphology.

### Histopathology findings and correlation to FFDM

As can be seen in Table 1, 8 (10.3%) of the 78 suspicious amorphous microcalcifications were classified as malignant-6 were DCIS (pB5) and 2 were on the borderline between DCIS and ADH (pB4)-malignancy being confirmed at surgery, and 36 (46.2%) were classified as benign (pB2). In addition, 34 (43.6%) of the 77 patients were diagnosed with pB3 lesions. Of those 34 lesions, 26 (76.4%) were precursor lesions-ADH (n = 14), LCIS (n = 2), ALH (n = 2), and FEA (n = 8)-and 8 (23.5%) were other high-risk lesions-RS (n = 7) and papillary lesion (n = 1).

**Table 1:** Distribution of suspicious amorphous microcalcifications, by pathological category and type of lesion, together with the primary and secondary (associated) diagnoses.

Pathological category, n (%)	Lesion type, n (%)	Number of cases, (%)	Primary diagnosis	Secondary diagnoses
B2, 36 (46.2)	Benign, 36 (46.2)	36 (46.2)	None	-
B3, 34 (43.6)	Precursor, 26 (33.3)	14 (17.9) 2 (2.6) 2 (2.6) 8 (10.3)	ADH LCIS ALH FEA	3 LCIS; 1 ALH; 7 FEA - 1 FEA -
	High-risk, 8 (10.3)	7 (9.0) 1 (1.3)	RS Papillary	- -
B4, 2 (2.6) B5, 6 (7.6)	Malignant, 8 (10.3)	2 (2.6) 6 (7.6)	DCIS* DCIS	- 1 ADH; 3 FEA; 1 ALH

* Borderline between ADH and DCIS on VABB; malignant at surgery.

### Surgical excision and patient follow-up

Surgical excision was performed in 18 (52.9%) of the 34 pB3 lesions, including17 precursor lesions- ADH (n = 10), LCIS (n = 1), ALH (n = 2), FEA (n = 4)-and 1 high-risk lesion-an RS. For the pB3 lesions, the underestimation rate was zero. Of the 34 patients with pB3 lesions, 16 (47.1%) were not submitted to surgical excision. All of those patients underwent mammographic follow-up. The mammographic follow-up of the 36 benign lesions and the 16 pB3 lesions not submitted to surgical excision showed no significant changes at the biopsy site. Follow-up was conducted for a minimum of 6 months and a maximum of 55 months (mean, 22 months).

Although the statistical analysis included only the primary diagnoses, a high number of secondary precursor lesions were identified in the VABB core samples. Of the 42 lesions classified as pB3, pB4, or pB5, 19 were precursor/high-risk lesions. Among the cases of DCIS, there were 5 secondary lesions: 1 ADH, 1 ALH, and 3 FEAs. Among the cases of ADH, secondary lesions were found in 11: ALH (n = 1); LCIS (n = 3); and FEA (n = 7). Among the cases of ALH, 3 secondary lesions (all FEA) were observed.

## DISCUSSION

Studies of screening programs have shown that cancer detection rates are higher for digital mammography than for conventional screen-film mammography^(12-14)^, especially among patients with microcalcifications^(4-6,13)^. However, such studies have evaluated microcalcifications of all morphologies together, despite the fact that different morphologies are known to be associated with different malignancy rates^(15,16)^.

Digital mammography has a higher detection rate for microcalcifications than does conventional mammography^(17,18)^. All of the images evaluated in the present study were obtained with FFDM, and we were therefore able to detect a large number of amorphous microcalcifications, because these are so small and/or hazy in appearance. The detection rate in our study was high in comparison with those reported in previous studies, which is probably due to the fact that our analysis was based solely on FFDM, whereas those of other studies have been either based solely on conventional mammography or based on a mixture of breast imaging techniques^(15,16,19-21)^, as well as because our study was conducted during the first year after the introduction of the digital technique into the breast cancer screening protocol of the clinics in question^(22)^.

We detected malignancy in 10.3% of the suspicious amorphous microcalcifications. The malignancy rates reported in the literature range from 13% to 31%, and, again, most of the studies evaluating such microcalcifications have employed conventional mammography^(16,19-21)^, as detailed in Table 2. The slight discrepancy between the malignancy detection rates observed in our study and those reported in the literature could be attributable to several factors: we evaluated solely FFDM images; we included microcalcifications with amorphous or punctate/amorphous morphology; and the time span for the evaluation of lesions differed. For instance, one study, based exclusively on conventional mammography, analyzed clustered amorphous calcifications that were not clearly stable for at least 5 years^(20)^.

**Table 2:** Comparison between the present study and others in the literature, in terms of the distribution of malignant lesions and precursor lesions.

			Malignant lesions			Precursor lesions	
Reference	Mammography technique	Number of cases	n	(%)		n	(%)
Present study	D	78	8	(10.3)		26*	(33.3)
Burnside et al.^(16)^	D and C	30	4	(13.3)		4^†^	(13.3)
Liberman et al.^(19)^	C	35	9	(25.7)		-	-
Berg et al.^(20)^	C	150	30	(20.0)		30^†^	(20.0)
Shin et al.^(21)^	C	100	31	(31.0)		8	(8.0)

D, digital; C, conventional.

* Including ADH, LCIS, ALH, and FEA;

† Including ADH, LCIS, and ALH.

Amorphous calcifications diagnosed on FFDM can represent calcifications in the initial stages of formation and might be related to slight changes, on the spectrum of modifications associated with the formation of cancer, and FFDM thus allows the detection of precursor lesions^(23)^. In the present FFDM study, suspicious amorphous microcalcifications correlated more often with precursor lesions than with malignant lesions (in 33.3% and 10.3% of cases, respectively).

Precursor lesions (including ADH, LCIS, and ALH) are detected in 8-20% of patients presenting with grouped amorphous calcifications^(16,20,21)^. In the present study, that rate was 23.1% (or 33.3% if FEAs are included), higher than the 2-15% reported in the literature for all suspicious breast lesions^(24)^. The higher rate observed in the present study might be attributable to the fact that we evaluated only amorphous calcifications detected on FFDM. Also, microcalcifications distributed in a cluster with amorphous morphology is the most frequent mammographic finding of FEA^(25)^. 

The high rate of detection of precursor lesions in comparison with that of detection of malignancy (33% vs. 10%), together with the fact that all cancers were DCIS and there was no underestimation of pB3 lesions, confirmed the correlation between suspicious amorphous calcifications on FFDM and early diagnosis. That was most notable for precursor lesions. Therefore, the diagnosis of a precursor lesion (excluding ADH, which can be classified as pB4 depending on the severity and extent of the lesion) in VABB core samples should not necessarily be considered indicative of underestimation of malignancy, because it could represent an appropriate diagnosis when the lesion is fully excised and correlated with cores containing calcifications. In addition to the fact that amorphous calcifications correlated more strongly with precursor lesions than with malignant lesions, we believe that the lack of underestimation of the malignancy of pB3 lesions might be due to the experience of the diagnostic team with VABB, which therefore yielded considerably fewer false-negative results^(26)^, as well as to the fact that a dedicated breast pathologist analyzed separate core vials, one containing microcalcifications and one containing other fragments^(27)^.

The diagnosis and management of high-risk breast lesions currently constitute a dilemma, especially because of recent improvements in detection^(26)^. The use of VABB to diagnose precursor lesions within amorphous calcifications seen on digital mammography allows us to identify patients at high risk for developing breast cancer, who could benefit from individualized preventive measures. In addition to special mammography screening, such patients could benefit from the use of annual magnetic resonance imaging scans, as per the recommendations of the American Cancer Society^(28)^. Other promising approaches include chemoprophylaxis^(29)^, as well as the more radical approach (prophylactic mastectomy) requested by some patients following the diagnosis of high-risk lesions. Therefore, mammography might serve not only as a form of secondary prevention of breast cancer but also as a primary preventive measure. By diagnosing precursor lesions, we can intervene in the disease process prior to the emergence of breast cancer.

In a study involving the use of VABB with an 11-gauge needle, Liberman et al.^(19)^ reported that the rate of non-retrieval of all calcifications was significantly higher for grouped amorphous calcifications than for all calcification morphologies, as it was for lesions smaller than 0.5 cm. However, it is important to attempt the retrieval of all calcifications during VABB. In our study, the underestimation rate was zero. That could be due to the great number of fragments we removed and to the presence of microcalcifications within those fragments. Jackman et al.^(30)^ reported underestimation rates of 8% when the entire lesion was removed, 13% when maximum lesion diameter was < 1.0 cm, 17% when calcifications were present, and 35% in the presence of mass lesions. In other words, the underestimation of calcifications was less than was that of mass lesions.

Our study has certain limitations. Our patient sample was small, and not all patients in whom surgical excision was recommended underwent the procedure. In addition, the follow-up period was, on average, relatively short. However, the bias was minimized by the fact that the group of patients studied was equivalent to the population that was excluded. Furthermore, interobserver variability is inherent in the practice of radiology. Moreover, there is no consensus on the use of the term amorphous, which could lead to differences among treatment centers in terms of the rates of detection and underestimation of malignancy^(31)^.

## CONCLUSION

Suspicious amorphous calcifications diagnosed on FFDM and submitted to VABB correlate strongly with precursor lesions. That knowledge should be taken into consideration in the management of the patients affected.

Studies seek to find parameters that facilitate the management of patients diagnosed with precursor lesions on percutaneous biopsy, informing decisions regarding the choice between surgery and follow-up alone. A multidisciplinary team can offer individualized treatment options for patients with concordant findings in the imaging and histological analyses^(32-34)^. Precursor lesions are of low grade, with a low risk for disease progression^(35)^. Therefore, with appropriate screening for high-risk patients, we believe that, in the event of disease progression, the diagnoses can still be made without affecting the prognosis.

Further studies involving larger samples and longer follow-up are needed. Approaches can be tailored on the basis of risk factors, patient age, the type/size of the lesion on imaging, histopathology, the extent of lesion excised, and the correlation with microcalcifications.
